# Activation of human neutrophils by *Esenbeckia leiocarpa*: comparison between the crude hydroalcoholic extract (CHE) and an alkaloid (Alk) fraction

**DOI:** 10.1186/1476-9255-9-19

**Published:** 2012-05-28

**Authors:** Rafael de Liz, Heros Horst, Moacir Geraldo Pizzolatti, Tânia Silvia Fröde, Denis Girard

**Affiliations:** 1 Laboratoire de recherche en inflammation et physiologie des granulocytes, Université du Québec, INRS-Institut Armand-Frappier, Laval, QC, Canada; 2 Department of chemistry, Center of Physical and Mathematical Sciences, Federal University of Santa Catarina, Campus Universitário, Trindade, 88040-970, Florianópolis, SC, Brazil; 3 Department of Clinical Analyses, Center of Health Sciences, Federal University of Santa Catarina, Campus Universitário, Trindade, 88040-970, Florianópolis, SC, Brazil; 4 INRS-Institut Armand-Frappier, 531 boul. des Prairies, Laval, QC, H7V 1B7, Canada

**Keywords:** Plant extracts, *Esenbeckia leiocarpa*, Inflammation, Neutrophils

## Abstract

*Esenbeckia leiocarpa*, a wide spread native Brazilian tree, was reported recently to possess anti-inflammatory effects in vivo, but the mechanisms involved are still not fully understood and its role in neutrophils is poorly documented. The aim of this study was to compare the effects of a crude hydroalcoholic extract (CHE) and an alkaloid-enriched (Alk) fraction obtained from *Esenbeckia leiocarpa* bark on human neutrophils by investigating the effect of each fraction alone or in a mixture with classical neutrophil agonists. CHE inhibited intracellular reactive oxygen species (ROS) production but increased the extracellular superoxide (O_2_^-^) production, while Alk increased the former and also slightly increased O_2_^-^ production. We found that CHE and Alk also induced phagocytosis accompanied by Syk activation, adhesion and degranulation. However, neither CHE nor Alk potentiated the effect of classical neutrophil agonists, namely the cytokines GM-CSF for phagocytosis and TNF-α for adhesion or N-formyl-methionyl-leucyl-phenylalanine (fMLP) for degranulation. In addition, based on catalase treatment, CHE and Alk induced neutrophil apoptosis by a hydrogen peroxide (H_2_O_2_)-dependent mechanism. Since the elimination of apoptotic neutrophils by professional phagocytes is important for the resolution of inflammation, the ability of CHE and Alk to induce neutrophil apoptosis has to be considered as one possible mechanism associated with the anti-inflammatory activity of these fractions previously reported in vivo.

## Background

*Esenbeckia* genus (Rutaceae) comprises a variety of species which have been popularly used to treat malaria in the Brazilian Amazon region [1,2]. Despite its ethnopharmacological approach, other biologic effects have been attributed to *Esenbeckia* species, including anticholinesterasic [3], antimicrobial [4], and antiparasitic [5] properties. *Esenbeckia leiocarpa* is a wide spread native Brazilian tree popularly known as guarantã, goiabeira, or guarataia [3]. Recent studies carried out in our laboratory have shown that this herb exerts a potent anti-inflammatory effect, including a decrease in leukocyte migration, exudate levels, and pro-inflammatory mediators in different *in vivo* models [6,7]. Several studies have singled out alkaloids as being the most abundant class of chemical compounds present in *Esenbeckia leiocarpa*[6-9]. Notwithstanding, these compounds are known for their anti-inflammatory activity. For instance, phenanthroindolizidine and septicine alkaloid have demonstrated *in vitro* anti-inflammatory properties, since they supressed nitric oxide production in RAW 264.7 cells stimulated with LPS and interferon-γ (IFN-γ) [10]. Other alkaloids, such as dehydroevodiamine, evodiamine, and rutaecarpine were effective in reducing both phorbol 12-myristate 13-acetate (PMA)- and N-formyl-methionyl-leucyl-phenylalanine (fMLP)-induced reactive oxygen species (ROS) production in neutrophils [11]. In addition, recent studies have shown that the alkaloid berberine induces apoptosis of human rheumatoid arthritis fibroblast-like synoviocytes [12].

Polymorphonuclear neutrophils (PMNs) have an essential role in the inflammatory response, since they are the first line of host defense against foreign microorganisms [13]. Notwithstanding, they have been implicated in the pathogenesis of several inflammatory diseases, including rheumatoid arthritis [14], type 2 diabetes [15], and chronic obstructive pulmonary disease [16]. These cells are activated and recruited to inflammation sites, where they exert phagocytic activity against invading pathogens and release different pro-inflammatory cytokines and chemokines, such as tumour necrosis factor-alpha (TNF-α) and interleukin-8 (IL-8) [17], as well as a variety of antimicrobial agents, including cationic peptides, proteases, lactoferrin, myeloperoxidase (MPO), and reactive oxygen species (ROS) by the exocytosis of cytoplasmic granules [13]. The aim of the present study was to compare the effects exerted by the crude hydroalcoholic extract and by an alkaloid-enriched fraction obtained from *Esenbeckia leiocarpa* bark, upon different neutrophil functions. Our results are the first to show that alkaloids represent an important fraction containing molecules responsible for the effect of *Esenbeckia leiocarpa* on phagocytosis, adhesion, and degranulation of human neutrophils, but not on ROS production.

## Methods

### Reagents

Dimethyl sulfoxide (DMSO), phorbol 12-myrostate 13-acetate (PMA), tumour necrosis factor-alpha (TNF-α), N-formyl-methionyl-leucyl-phenylalanine (fMLP), and lipopolysaccharide (LPS) were purchased from Sigma-Aldrich (St. Louis, MO, USA). Granulocyte macrophage colony-stimulating factor (GM-CSF) was purchased from Pepro Tech Inc. (Rocky Hill, NJ, USA). The monoclonal antibodies against CD35 (clone E11) and CD63 (clone H5C6) were purchased from BD PharMingen (San Diego, CA, USA). The anti-CD66b mAb (clone 80H3) was obtained from AbDSerotec (Raleigh, NC, USA). The Syk inhibitor II (catalog no.57472) was purchased from EMD Biosciences. Specific rabbit-anti-human phosphorylated Syk antibody was purchased from Cell Signaling Technology (Danvers, MA, USA). Specific mouse Ab anti-human Syk was purchased from Santa Cruz Biotechnology (Santa Cruz, CA, USA).

### Plant materials, preparation of crude hydroalcoholic extract and alkaloid fraction

Various samples of bark from *Esenbeckia leiocarpa* were collected in Arenápolis, a town located in the state of Mato Grosso, Brazil. They were collected in August 2007 and were identified by Dr. Celice Alexandre, of the University of the State of Mato Grosso, Tangará da Serra, MT, Brazil, where a voucher specimen (38639) was deposited. Dried *Esenbeckia leiocarpa* bark (9 kg) was macerated and extracted with 90% EtOH (24 h × 3) resulting in a crude hydroalcoholic extract (CHE) (290 g). Some of the CHE (20 g) was partitioned between EtOAc and 5% HCl solution. The pH of the acidic water-soluble material was adjusted to pH 9–10 with 10% ammonia solution and was then extracted with EtOAc to yield an alkaloid (Alk) fraction (5g) [6,7]. Based on preliminary results, CHE and Alk were used at concentrations of 500 and 100 μg/mL, respectively; at these concentrations, cell necrosis never exceeded 5%, as assessed by trypan blue exclusion assay, and close to 80% of cells were in apoptosis (*data not shown*).

### Neutrophil isolation

Neutrophils were isolated from venous blood of healthy volunteers by dextran sedimentation, followed by centrifugation over Ficoll-Hypaque (Amersham Pharmacia Biotech Inc., Baie d’Urfé, Québec, Canada), as described previously [18]. Blood donations were obtained from informed and consenting individuals according to institutionally approved procedures. Cell viability was monitored by trypan blue exclusion and found to be consistently >98%. Cell purity (>98%) was verified by cytology from cytocentrifuged preparations coloured by Hema-3 staining kit (Biochemical Sciences Inc., Swedesboro, NJ, USA). Cell viability was evaluated systematically before and after each treatment. Neutrophils were then resuspended (10×10^6^ cells/mL in RPMI-HEPES medium (25 mM), supplemented with penicillin (100 U/mL)/streptomycin (100 μg/mL)) for all experiments.

### Measurement of intracellular ROS production

To determine the effects of CHE and Alk on intracellular levels of ROS, cells (1 × 10^6^ cells/mL) were incubated for 5, 15, or 30 min with either CHE (500 μg/mL) or Alk (100 μg/mL) in the presence or absence of phorbol 12-myristate 13-acetate (PMA). Intracellular levels of ROS were detected using the probe 2′,7′- dichlorofluorescein diacetate (H_2_DCFDA; Molecular Probes, Eugene, OR, USA), as per the manufacturer’s recommendation. After stimulation, cells were washed with PBS and stained with non-fluorescent cell-permeable H_2_DCFDA (5 μg/mL) for 15 min at 37°C. The H_2_-dichlorofluorescein oxidizes rapidly to the highly fluorescent dichlorofluorescein by ROS. As a positive control, the fluorescence intensity of cells pre-treated with H_2_DCFDA was measured in the presence of PMA (10^−7^ M).

### Phagocytosis of sheep erythrocytes

Sheep red blood cells (SRBCs) were opsonized with a final 1/200 dilution of rabbit IgG anti-SRBC antibody (Sigma-Aldrich) by incubation for 45 min at 37°C, as previously described [19,20]. PMNs (4 × 10^6^ cells/mL in RPMI 1640) were pre-treated 30 min with buffer (1% DMSO), GM-CSF (65 ng/mL) (positive control), as well as with CHE (500 μg/mL) or Alk (100 μg/mL), in the presence or absence of GM-CSF (65 ng/mL). Cells were also incubated in the presence or absence of two different Syk inhibitors, piceatannol (30 μM) or Syk inhibitor II (30 μM). PMNs were then incubated with 20 × 10^6^ opsonized SRBCs for 45 min as described above. The samples were centrifuged at 200 × *g* at 4°C for 10 min. Supernatants were discarded, and non-ingested SRBCs were eliminated by performing osmotic shock on the pellets, by treating them with 300 μL H_2_O for 15 s followed immediately by the addition of 4.5 mL ice-cold PBS (PBS; 1×). The samples were washed twice with ice-cold PBS, and the pellets were suspended to a final concentration of 4 × 10^6^ cells/mL. Duplicate cytocentrifuged preparations were prepared in aliquots of ~200 μL, stained with the Hema-3 staining kit (Biochemical Sciences Inc., Swedesboro, NJ, USA), and observed by cytology, essentially as previously documented [19,21]. Phagocytosis was measured as percentage of neutrophils ingesting at least one opsonized SRBC.

### Neutrophil adhesion assay

The adhesion assay was performed essentially as previously documented [22]. The human epithelial lung cell line A549 (ATCC) was grown in RPMI 1640 supplemented with 10% FCS and antibiotics. Cell viability was systematically evaluated before and after each treatment, and mortality never exceeded 5%. A549 cells were grown on glass coverslips; when at confluence, they were washed twice with PBS. PMNs were pre-treated for 30 min with buffer (DMSO 1%) with or without either CHE (500 μg/mL) or Alk (100 μg/mL) in the presence or absence of tumour necrosis factor-alpha (TNF-α) (10 ng/mL), and were labelled for 30 min with 5 μM calcein-AM (Molecular Probes, Eugene, OR, USA), according to the manufacturer’s recommendation. After incubation, 500 μL of neutrophil suspension (10 × 10^6^ cells/mL) was added to each well of a 12-well plate containing confluent A549 cells on a coverslip for 30 min. After the incubation, coverslips were extensively washed and mounted on a glass slide. The number of adherent PMNs was calculated by counting the number of fluorescent cells from five randomly selected high-power fields (400x) observed with a photomicroscope Leica equipped with an ebq 100 dc epifluorescent condenser.

### Degranulation of human neutrophils

Cell surface expression of CD35, CD63, and CD66b was monitored for assessing degranulation of secretory, azurophilic, and specific granules, respectively, by flow cytometry, as previously described [23,24]. Briefly, non-specific binding of the antibodies was prevented by incubating the cells with PBS + 20% decomplemented autologous serum for 30 min on ice. After several washes with PBS, primary antibodies or an IgG1 isotype control antibody were added at a concentration of 1 μg/mL in PBS for 30 min on ice. Cells were washed three times in PBS and incubated with 1 μg/mL FITC-conjugated goat anti-mouse IgG antibody for 30 min on ice. After three washes, cells were resuspended in PBS, and analysis was performed with a FACScan (BD Biosciences, San Jose, CA, USA).

### Zymography assay

Neutrophils (1 × 10^6^ cells/mL in RPMI 1640 per condition) were pre-treated 30 min with buffer (1% DMSO), with or without CHE (500 μg/mL) or Alk (100 μg/mL), in the presence or absence of LPS (1 μg/mL), and then centrifuged at 13,000 rpm for 10 min at 4°C, and the pellets were discarded. The supernatants (5 μL corresponding to 50,000 cells) were mixed with a non-reducing buffer (40% glycerol, Tris–HCl 1 M, pH 6.8, SDS 8%) and separated on 7.5% acrylamide gels containing 0.2% gelatin. Gels were washed twice for 30 min with 2.5% Triton X-100 and incubated overnight in digestion buffer (Tris–HCl 50 mM, pH 7.4, NaCl 150 mM, CaCl_2_ 5 mM). Gels were stained with Coomassie blue 0.1% and then destained.

### Assessment of neutrophils apoptosis by cytology

Assessment of neutrophil apoptosis was performed essentially as previously described [25]. Briefly, freshly isolated human neutrophils (10 × 10^6^ cells/mL in RPMI 1640 supplemented with 10% autologous serum) were incubated for 24h in the presence (+) or absence (−) of CHE, Alk, with or without catalase. Cytocentrifuged preparations of neutrophils were performed with a Cyto-tek centrifuge (Miles Scientific, Elkart, IN, USA), as previously described and then were stained with the Hema-3 staining kit, as per the manufacturer’s protocol. Cells were examined by light microscopy at a final magnification of 400X and apoptotic neutrophils were defined as cells containing one or more characteristically dark-stained pyknotic nuclei. Results were expressed as percentage of cells in apoptosis.

### Western blot analysis

Neutrophils (10 × 10^6^ cells/mL in RPMI-HEPES (25 mM), penicillin (100 U/mL)/streptomycin (100 μg/mL)) were stimulated with GM-CSF (65 ng/mL), CHE (500 μg/mL, Alk (100 μg/mL), or buffer (DMSO 1%) for 30 min at 37°C. At the end of the incubation period, the cells were lysed in 4x Laemmli’s sample buffer (0.25 M Tris–HCl, pH 6.8, 8% SDS, 40% glycerol, and 20% 2β-ME), and aliquots corresponding to 1 × 10^6^ cells were loaded onto 10% SDS-PAGE and transferred to nitrocellulose membranes (Amersham Pharmacia Bio-tech Inc., Baie d’Urfé, Que, Canada). Non-specific sites were blocked with 3% bovine serum albumin (BSA) in TBS-Tween (25 mMTris-HCl, pH 7.8, 190 mMNaCl, 0.15% Tween-20), and blots were incubated with antibodies as previously described [19]. Monoclonal rabbit anti-human phosphorylated Syk antibody (1:1000; Danvers, MA, USA) and HRP-goat anti-rabbit (1:15,000) were used. Membranes were stripped for 30 min at 55°C with stripping buffer (100 mM 2-ME, 2% SDS, 62.5 mM Tris, pH 6.7), washed, and reprobed with an specific mouse Ab anti-human Syk (1:1000; Santa Cruz, CA, USA) followed by a HRP-conjugated goat anti-mouse IgG + IgM (1:20,000; Jackson ImmunoResearch Laboratories, Inc.). Syk protein expression was revealed with ECL as per manufacturer’s instructions.

### Superoxide production

O_2_^-^ production was performed by colorimetric assay (reduction of cytochrome c), as previously published^25^. Briefly, neutrophils (1 × 10^6^ cells/mL in Hank’s balanced salt solution (HBSS) supplemented with 1.6 M CaCl_2_) were incubated with 130 μ cytochrome c (Sigma-Aldrich) for 5 min at 37°C in the presence (+) or absence (−) of PMA, CHE or Alk. The absorbance of cytochrome c was monitored at 550 nm and the number of O_2_^.-^ anions produced was calculated as previously published [25]using an extinction coefficient of 21.1.

### Statistical analysis

Experimental data were expressed as mean ± SEM. Significant differences between groups were determined by Student’s *t* test, when necessary, or two-way-analysis of variance (two-way-ANOVA), and then performed using GraphPad Prism, Version 5.01. Differences were considered statistically significant as follows: *, *P* < 0.05 versus Ctrl or appropriated diluent.

## Results and discussion

### CHE is an inhibitor and Alk an inducer of ROS production in human neutrophils

**Figure 1:**
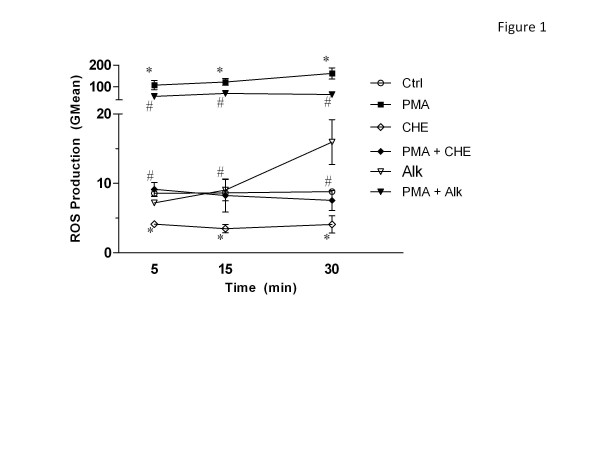
**Effect of CHE and Alk on intracellular ROS production in human neutrophils.** Freshly isolated human PMNs (1 x 10^6^ cells/mL) were incubated with diluent (1% DMSO in HBSS, Ctrl), the positive control, PMA, CHE (500 μg/mL), Alk (100 μg/mL), PMA + CHE or PMA + Alk for 5, 15 or 30 min and intracellular ROS production was assessed by flow cytometry as described in Materials. Results are means ± SEM (n = 4). *, *p* < 0.05 vs Ctrl; #, *p* < 0.05 vs PMA by student-*t* test.

As illustrated in Figure 1, CHE rapidly and significantly decreased the basal level of ROS production observed after 5 min of treatment. This decrease persisted for up to 30 min, as assessed by flow cytometry with the H_2_DCFDA fluorescent probe. Unlike CHE, Alk did not significantly decrease basal ROS production but, rather, induced it, although not significantly, after 30 min of incubation. ROS production was also determined in PMA-induced PMNs and both CHE and Alk caused significant decreases in ROS production.

### Modulatory activity of CHE and Alk on the ability of PMNs to exert phagocytosis, adhesion and degranulation

**Figure 2:**
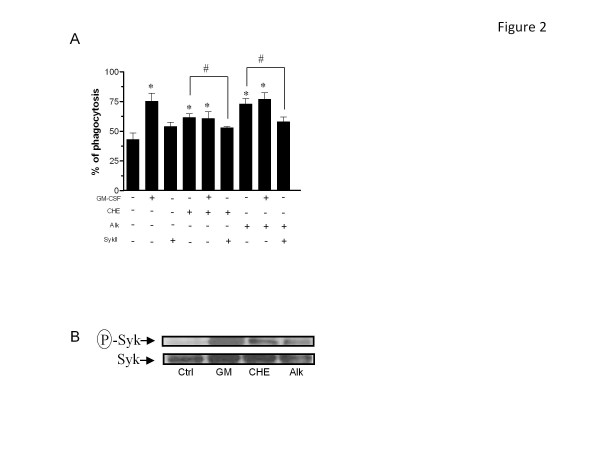
**CHE and Alk enhance Fc-mediated phagocytosis by a Syk-dependent mechanism.** PMNs were isolated an incubated in the presence (+) or absence (−) of GM-CSF, CHE, Alk or Syk II inhibitor (SykII). Phagocytosis of opsonized SRBCs **(A)** or Syk activation **(B)** were determined as described in Methods. A, results are means ± SEM (n = 4). *, *p* < 0.05 vs Ctrl (GM-CSF, CHE, Alk or SykII) and #, *p* < 0.05 vs GM-CSF for the indicated columns. B, upper panel is the phosphorylated form of Syk, whereas the bottom panel illustrated the non-phosphorylated form of Syk, following stripping indicating equal protein loading. Results are from one representative experiment out of three.

**Figure 3:**
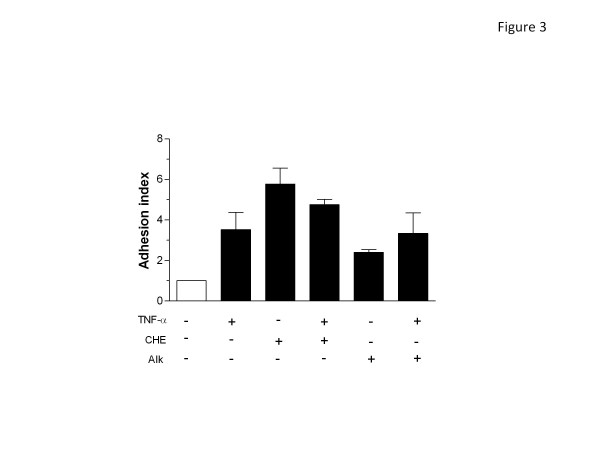
**CHE and Alk enhance neutrophil adhesion onto cell substratum.** PMNs were incubated with or without CHE, Alk, TNF-α, CHE + TNF-α or Alk + TNF-α for 30 min, labelled with calcein-AM, incubated on confluent A549 cells for 30 min and adhesion was measured as described in Methods. Results are means ± SEM (n = 4) and are expressed as indices, one being the basal or control level.

**Figure 4:**
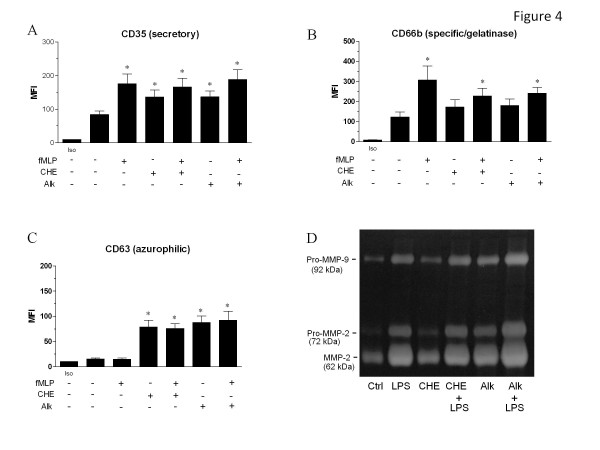
**CHE and Alk induce degranulation in human neutrophils.** PMNs were incubated in the presence (+) or absence of fMLP, CHE or Alk and the release of subset types of granules was evaluated by flow cytometry by monitoring cell surface expression of CD35 **(A)**, CD66b **(B)** or CD63 **(C)**, as described in Methods. Results are means ± SEM (n = 4). In panel **D**, supernatants from PMNs incubated with buffer (Ctrl), LPS, CHE, CHE + LPS, Alk, or Alk + LPS were collected and tested by zymography in order to determine whether an enzymatic activity is present, as described in Methods. Results are from one representative experiment out of four.

Given that CHE and Alk can modulate a rapid response such as ROS production in human PMNs, we then determined whether or not one or both fraction(s) could modulate phagocytosis, adhesion and degranulation, three major functions of PMNs important for host defense that occurs relatively rapidly. Therefore, we investigated the potential modulatory activity of CHE and Alk on these responses. As illustrated in Figure 2A, CHE and Alk significantly enhanced Fc-mediated phagocytosis of opsonized red blood cells (61.7 ± 3.2% and 73.0 ± 4.6%, respectively (n = 4)) when compared to untreated cells (43.0 ± 5.6%), but did not potentiate the effect of the cytokine GM-CSF. In addition, this figure shows that the ability of either CHE or Alk to enhance phagocytosis was reversed by a Syk-selective inhibitor, indicating that both fractions enhanced phagocytosis by a Syk-dependent mechanism. To further elucidate this, we then demonstrated that Syk was activated in response to CHE or Alk, as evidenced by its increased level of phosphorylation (Figure 2B). CHE markedly increased the adhesion of PMNs on human epithelial A549 cells (ratio of 5.8 ± 0.8, n = 3); (Figure 3) this increased adhesion was even greater than that of the potent cytokine TNF-α (3.5 ± 0.9) used as a positive control in this assay [22]. However, when PMNs were treated simultaneously with CHE and TNF-α, the resulting ratio was 4.8 ±0.3, which indicated an effect intermediate between that produced by either CHE or TNF-α only. Alk also increased the adhesion of PMNs but to a lesser extent than CHE (2.4 ±0.1), but when mixed with TNF-α, the result was similar to that of PMNs treated with TNF-α only (3.3 ± 1.0). We then evaluated the ability of CHE and Alk to modulate degranulation in neutrophils as assessed by flow cytometry, by monitoring cell surface expression of three important molecules known to be present in the different types of granule: CD63 (azurophilic granules), CD66b (specific and gelatinase granules) and CD35 (secretory granules) [24,26]. As illustrated in Figure 4A, CHE and Alk induced, with the same potency, the degranulation of secretory granules, as evidenced by the increased cell surface expression of CD35, although to a lesser extent than fMLP, used as positive control. The cell surface expression of CD35 was not significantly modulated when cells were treated with CHE + fMLP and Alk + fMLP, when compared with fMLP only. CHE and Alk increased cell surface expression of CD66b but, unlike for CD35, when CHE or Alk was mixed with fMLP, the expression of CD66b was slightly decreased when compared to that produced by fMLP only (Figure 4B). Cells surface expression of CD63 remained stable in response to fMLP when compared to control cells, but was markedly, and significantly, increased by CHE and Alk (Figure 4C). In order to support CHE- or Alk-induced degranulation based on cell surface expression of a given marker, we then used the supernatants and performed zymography experiments. As illustrated in Figure 4D, CHE and Alk increased the activity of the matrix metalloproteinase-9 (MMP-9), also known as 92 kDa gelatinase (gelatinase B), and MMP-2 or 72 kDa gelatinase (gelatinase A), as evidenced by an increased expression of the digested regions (bands) in the gel. Of note, gelatinase activity was more pronounced in response to Alk when compared to CHE. Also, Alk potentiated the activity of LPS, whereas CHE did not (or only slightly) promote such activity.

### Inhibition of H_2_O_2_ reverses the ability of CHE and Alk to accelerate apoptosis

**Figure 5:**
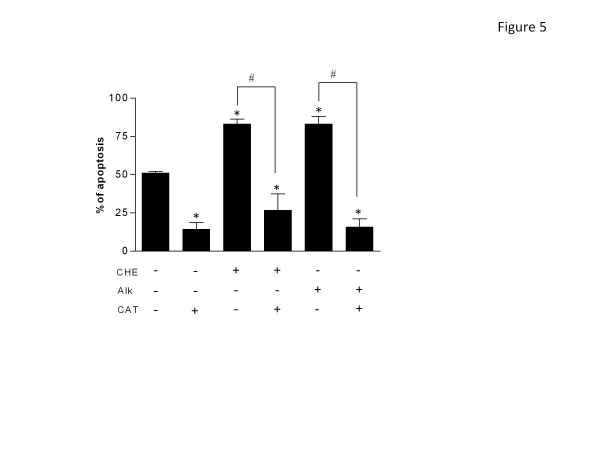
**CHE and Alk induce apoptosis in human PMNs by a H**_**2**_**O**_**2**_**-dependent mechanism.** PMNs were isolated and incubated (10 × 10^6^ cells/mL) with buffer, CHE (500 μg/mL) or Alk (100 μg/mL) in the presence (+) or absence (−) of catalase (CAT) for 24 h. Apoptosis was evaluated by cytology as described in Methods. Results are means ± SEM (n ≥ 3). *, *p* < 0.05 vs Ctrl (CHE, Alk and CAT), #, *p* < 0.05 vs CHE or Alk (as indicated) by Student’s *t*-test.

Neutrophils are known to undergo spontaneously in apoptosis (SA) [27] and many agents can accelerate or delay this biological process [25,28,29]. As illustrated in Figure 5 both CHE and Alk fractions accelerated SA. As expected, addition of catalase, an enzyme that degrades hydrogen peroxide (H_2_O_2_), significantly delayed SA [25] and, also prevented the ability of CHE and Alk to accelerate SA. This suggested that CHE and Alk can also modulate a PMN function requiring several hours of incubation *in vitro* (24h).

### CHE and Alk increase extracellular O_2_^-^ production in PMNs

**Figure 6:**
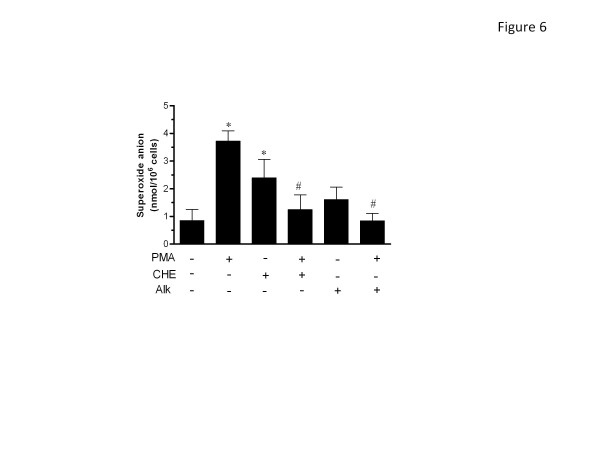
**Effect of CHE and Alk on superoxide production.** PMNs were incubated in the presence (+) or absence (−) of PMA, CHE or Alk and O_2_^.-^ production was determined after 5 min as described in Methods. Results are means ± SEM (n = 3). *, *p* < 0.05 vs Ctrl (no CHE, Alk or PMA); #, *p* < 0.05 vs PMA, by Student’s *t*-test.

Because treatment with catalase inhibited the capacity of CHE and Alk to induce apoptosis, and since catalase used in the experiments is not cell permeable and degraded therefore H_2_O_2_ present in the extracellular medium, we then decided to verify whether extracellular ROS production was also increased in response to CHE and Alk. We measured the classic response of O_2_^-^ production, since O_2_^-^ anions are rapidly transformed into H_2_O_2_ via the action of the enzyme superoxide dismutase (SOD). As illustrated in Figure 6, after only 5 min of treatment, CHE significantly increased the rapid production of O_2_^-^, whereas Alk did not, although a slight tendency towards increased production was noted. However, both CHE and Alk were found to significantly reduce the PMA-induced O_2_^-^ production.

## Discussion

The discovery that *Esenbeckia leiocarpa* possesses some anti-inflammatory properties *in vivo* is recent [6,7]. The present work is the first study to investigate, in parallel, the role of both the crude hydroalcoholic extract (CHE) and an alkaloid fraction (Alk), prepared from *Esenbeckia leiocarpa* bark in human PMNs, key players in inflammation. The various PMN functions were tested, not only in CHE- or in Alk-stimulated cells, but also, in cells treated with a combination of CHE or Alk and classical PMN agonists, namely, PMA for ROS production, GM-CSF for phagocytosis, TNF-α for adhesion, and fMLP for degranulation. This strategy allowed us to study not only the direct effect of CHE or Alk on PMNs, but also when mixed with classical neutrophil agonists, a situation that is likely to occur *in vivo* where PMNs could be activated by several agents during inflammation [30]. From our results, it is clear that either CHE or Alk showed agonistic activities for human PMNs. This is consistent with a previous study indicating that *E. Leiocarpa* exerted an important anti-inflammatory effect *in vivo* in neutrophils, since a decrease of myeloperoxidase was noted [6], an enzyme considered as an important marker of PMN activation [31]. In general, for all tested functions, PMNs responded to both CHE and Alk in a similar fashion, except for ROS production; CHE inhibited intracellular ROS production but increased the extracellular O_2_^-^ production, whereas Alk (moderately) increased the former and also increased O_2_^-^ production, but not significantly. Of interest, both the crude extract and the alkaloid-enriched fraction also demonstrated similar effects *in vivo*; they were found to inhibit leukocyte migration, exudate concentrations and proinflammatory mediators in carrageenan-induced inflammation in the murine air pouch model [6]. Therefore, Alk possesses some effects different than CHE *in vitro* and *in vivo*. It is difficult to explain with certainty why CHE decreased intracellular ROS production but increased extracellular O_2_^-^ production, while Alk (slightly to moderately) increased both of these responses. One of the most probable reasons, although purely speculative at the moment, is the presence of other chemical compounds in the CHE, apart from alkaloids that are equally present in the Alk. Of interest, CHE and Alk increased the PMN apoptotic rate to almost the same level after 24h and this response was reversed by catalase. This effect suggests that even if CHE and Alk do not exert a similar effect upon general ROS production (intra and/or extracellular), similar levels of H_2_O_2_ are eventually present in the external medium of CHE- or Alk-stimulated PMNs. Interestingly, the role of H_2_O_2_ has been recently determined during neutrophilic inflammation *in vivo* during antigen-induced arthritis[32]. In this study, neutrophil recruitment was delayed in gp91^*phox*^ KO mice (that do not generate ROS, at least via NADPH activation) or after administration of catalase but was resolved after administration of H_2_O_2_ or superoxide dismutase [32]. Given the general involvement of ROS during PMN apoptosis, especially the role of extracellular H_2_O_2_ (since addition of catalase almost completely reversed spontaneous or agent-induced apoptosis [25]), the ability of CHE and Alk to produce extracellular O_2_^-^, rapidly transformed into H_2_O_2_, is in accordance with the anti-inflammatory properties observed *in vivo*, since both CHE and Alk are proapoptotic for PMNs, an important event involved in the resolution of inflammation.

For the other functions tested, the difference observed between CHE and Alk was in terms of intensity of response. The most potent *in vitro* activity of CHE, when compared to Alk, is its ability to increase the adhesion of PMNs onto epithelial A549 cells. Whether CHE increased cell surface expression of some adhesion molecules, not induced by Alk, or if it acts by increasing such molecule expression with greater intensity than Alk in PMNs remains to be determined. The most potent *in vitro* activity of Alk when compared to CHE is its ability to strongly increase a gelatinase activity present in the supernatants, as assessed by zymography. Although this could not be directly related to the degranulation of gelatinase or specific granules (the cell surface expression of the CD66b marker is similar in CHE- or Alk-induced PMNs), it is important to specify that other enzymes also possess gelatinolytic activity [33] and this could, at least partially, explain why Alk and LPS had an increased gelatinolytic activity when compared to CHE. The ability of CHE and Alk to enhance Fc-mediated phagocytosis of opsonized SRBCs was, as other PMN agonists, including cytokines [19,20], linked to Syk activation.

## Abbreviations

CHE: Crude hydroalcoholic extract (CHE); Alk: Alkaloid Fraction Alk; ROS: Reactive oxygen species; O2-: Superoxide; H2O2: Hydrogen peroxide; GM-CSF: Granulocyte macrophage colony-stimulating factor; PMA: Phorbol 12-myristate 13-acetate; fMLP: N-formyl-methionyl-leucyl-phenylalanine; PMNs: Polymorphonuclear neutrophils; TNF-α: Tumour necrosis factor-alpha; SA: Spontaneous apoptosis.

## Competing interest

The authors have no competing interest.

## Authors’ contributions

RL carried out all experiments and participated in the data analysis and writing of the manuscript. HH and MGP were responsible for the plant extract preparations. TSF and DG, conceived of the study, participated in the design and coordination of the manuscript. All authors read and approved the final manuscript.
